# A novel de novo *Myocilin* variant in a patient with sporadic juvenile open angle glaucoma

**DOI:** 10.1186/s12881-016-0291-5

**Published:** 2016-04-14

**Authors:** Emmanuelle Souzeau, Kathryn P. Burdon, Bronwyn Ridge, Andrew Dubowsky, Jonathan B. Ruddle, Jamie E. Craig

**Affiliations:** Department of Ophthalmology, Flinders University, Flinders Medical Centre, Adelaide, Australia; Menzies Institute for Medical Research, University of Tasmania, Hobart, Australia; SA Pathology, Flinders Medical Centre, Adelaide, Australia; Centre for Eye Research Australia, University of Melbourne, Royal Victorian Eye & Ear Hospital, Melbourne, Australia

**Keywords:** De novo variant, Juvenile open angle glaucoma, Genetic testing, Glaucoma, Myocilin

## Abstract

**Background:**

Glaucoma is a leading cause of irreversible blindness. Pathogenic variants in the *Myocilin* gene (*MYOC*) cause juvenile open angle glaucoma (JOAG) in 8–36 % of cases, and display an autosomal dominant inheritance with high penetrance. Molecular diagnosis is important for early identification as therapies are effective in minimizing vision loss and *MYOC* variants can be associated to severe glaucoma. *MYOC* variants are usually inherited, however a fifth of carriers do not report a family history. The occurrence of *de novo MYOC* variants is currently unknown.

**Case presentation:**

In this study we investigated a 14 year old male Caucasian patient diagnosed with JOAG, and no family history of glaucoma. A novel probably deleterious MYOC:p.(Pro254Leu) variant was identified in the index case. This variant was not present in the parents or the siblings.

**Conclusion:**

This is the second report of a *de novo MYOC* variant in a sporadic case of JOAG and it is currently unknown if this mechanism occurs more frequently. This finding emphasizes the importance of screening individuals with JOAG for *MYOC* mutations irrespective of a negative family history.

## Background

Glaucoma is one of the leading causes of irreversible blindness affecting over 60 million individuals worldwide [1]. Primary open angle glaucoma (POAG, MIM 137760) is the most common type and is characterized by changes in the optic nerve head with corresponding visual field loss in the presence of an open anterior chamber angle [2]. Juvenile open angle glaucoma (JOAG) refers to a younger age at diagnosis usually defined by an onset before 30–40 years old and associated with a more severe phenotype [3, 4]. Therapies for POAG aim at controlling intraocular pressure (IOP) and are usually effective in minimizing disease progression [5–7]. However, the early stages are often asymptomatic and half of the cases remain undiagnosed, making it challenging to implement treatment before irreversible vision loss occurs.

Pathogenic sequence variants in the *MYOC* gene (MIM 601652) have been first described in association with JOAG in 1997 [8]. Since then, they have been consistently identified in 2–4 % of adult-onset POAG [9, 10] and in 8–36 % of JOAG [9, 11, 12] among different ethnicities. *MYOC* comprises three exons which encode a protein consisting of two major domains, an N-terminal myosin-like domain and a C-terminal olfactomedin-like domain [13]. Most disease causing variants are clustered within exon 3 in the olfactomedin domain [14]. The pathophysiology is not fully understood but it has been postulated that the accumulation of misfolded proteins lead to endoplasmic reticulum stress, which compromises the trabecular meshwork cells regulating the IOP [15]. *MYOC* pathogenic variants are inherited in an autosomal dominant fashion and are often associated with high IOP, younger age at diagnosis and strong family history and can result in severe glaucoma and blindness if left untreated [9, 10, 16].

The majority of *MYOC* carriers report a family history of glaucoma, however sporadic cases still account for 20 % of mutation carriers [9]. It is currently unknown whether sporadic cases could be explained by *de novo* variants. In this study, we report a JOAG sporadic case with a novel *de novo MYOC* variant, and discuss the occurrence of *de novo* variants in *MYOC* associated glaucoma and the implications for the patient and his family.

## Case presentation

### Clinical presentation

**Fig. 1:**
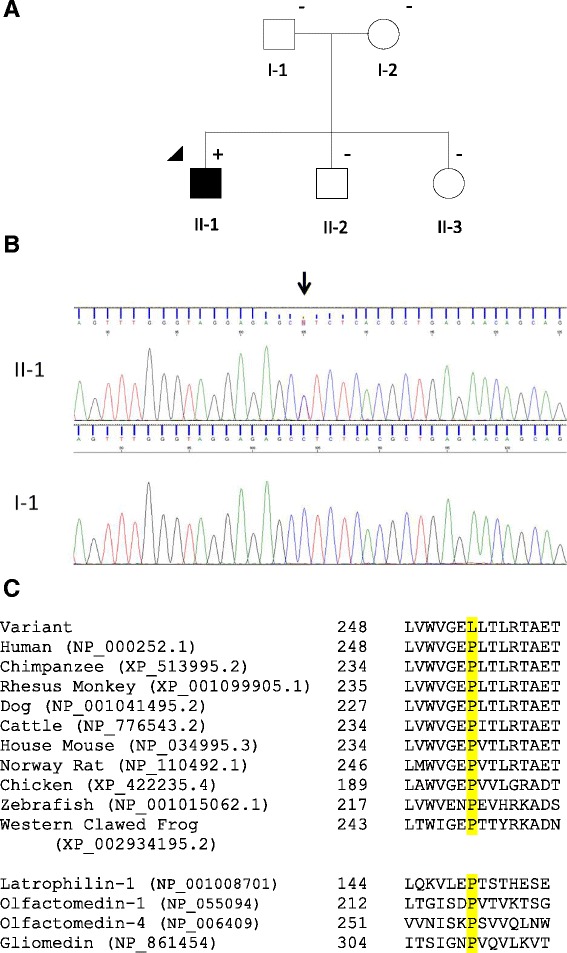
Pedigree and genetic analysis. **a** Pedigree of the family. Round symbols indicate female; square symbols, male; fully filled symbols, open angle glaucoma; unfilled symbols, unaffected; arrow, proband; plus/minus, presence/absence of the MYOC:p.(Pro254Leu) variant. **b** Chromatogram showing the presence of MYOC:c.761C > T, p.(Pro254Leu) sequence variant in individual II-1 at the top (affected) and its absence in individual I-1 at the bottom (unaffected). The black arrow marks the heterozygous variant. **c**. Alignment of MYOC protein sequences corresponding to residues 248 through 262 (NP_000252.1), against different species, and of different human olfactomedin proteins. The residue of interest, p.(Pro254Leu), is highlighted in yellow. Reference sequences IDs of the genes/species aligned are shown in brackets

**Fig. 2:**
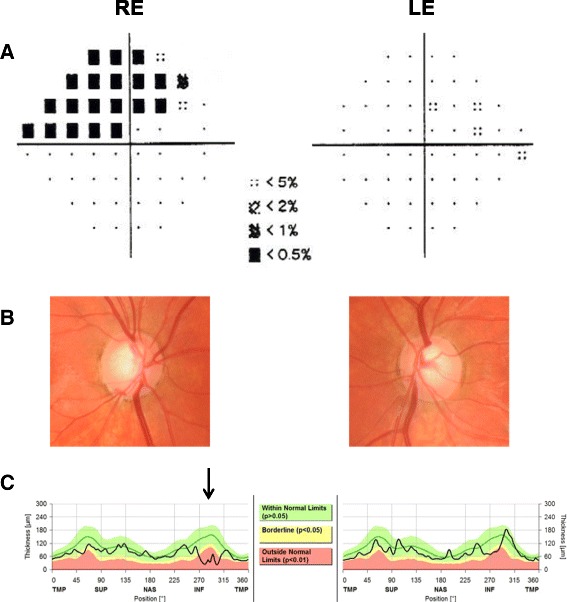
Clinical presentation of the index case. Glaucomatous defects in index case. **a** Visual field pattern deviation showing a superior arcuate defect involving fixation in the right eye (Humphrey Field analyser, Zeiss). **b** Optic discs photos showing a right infratemporal notch and disc haemorrhage. **c** Optical coherence tomography showing inferior retinal nerve fibre layer loss more prominent in the right eye than the left as shown by the black arrow (Spectralis®, Heidelberg Engineering). RE: right eye, LE: left eye, TMP: temporal, SUP: superior, NAS: nasal, INF: inferior

The pedigree of the family is shown in Fig. 1a. The index case and his family were referred to the Australian and New Zealand Registry of Advanced Glaucoma (ANZRAG) through his treating ophthalmologist [17]. The proband was a 14 year old Caucasian male patient (II-1). He was referred to an ophthalmologist following a routine optometrist review for his glasses prescription which revealed high IOP. Following examination, he was diagnosed with JOAG. His IOP at presentation were 31 mmHg in the right eye and 32 mmHg in the left. His vertical cup-to-disc ratio was 0.85 right and 0.8 left, and he had central field loss involving fixation in the right eye (Humphrey Field Analyzer, Zeiss) (Fig. 2a). His visual acuity was 20/20 in both eyes. His IOP was initially under control with latanoprost and brimonidine/timolol. However he underwent bilateral trabeculectomies following his most recent IOP which were 40 mmHg. Optic nerve appearances and retinal nerve fiber layer loss (Spectralis®, Heidelberg Engineering) are depicted in Fig. 2b and c. His parents and two siblings had normal eye examinations.

### Genetic testing

Genetic testing was performed through the National Association of Testing Authorities (NATA) accredited laboratories of SA Pathology at the Flinders Medical Centre in Adelaide, Australia. The proband was sequenced for the 3 coding exons of the *MYOC* gene as previously described [9]. A heterozygous substitution of Thymine for Cytosine at nucleotide 761 of the *MYOC* exon 3 coding sequence was identified (MYOC:c.761C > T), encoding a missense substitution of Proline to Leucine at position 254 (p.(Pro254Leu)) (Fig. 1b). No other variants were identified in the *MYOC* gene of the proband. JOAG can also be associated with *CYP1B1* variants [18]. The coding region of the *CYP1B1* gene was sequenced to exclude other causative genes. No disease-causing variants were identified in *CYP1B1*.

The p.(Pro254Leu) variant is novel since it was absent from the *MYOC* Database (www.myocilin.com), NCBI dbSNP (www.ncbi.nlm.nih.gov/SNP/), and the Exome Aggregation Consortium (http://exac.broadinstitute.org/) which comprises exome sequence data spanning 60 706 unrelated individuals. A search of the scientific literature also failed to identify any reference to this variant. However, a recent study reported a *MYOC* variant at the same residue p.(Pro254Arg) in a patient with JOAG and his affected mother [19]. SIFT and Polyphen-2 both predicted this variant to be deleterious, with sequence alignment demonstrating this position to be highly conserved among vertebrates and other olfactomedin domain-containing proteins (Fig. 1c). *MYOC* is a well characterized gene and codon position 254 resides in the core hydrophobic β-sheet belt of the olfactomedin domain, which is important in protein-protein interactions and is sensitive to aggregation in the presence of substitutions [20]. The p.(Pro254Leu) variant is likely pathogenic based on bioinformatics prediction, invariant conservation of this residue, and characterization of the protein structure. *MYOC* disease-causing variants can be associated with severe glaucoma and blindness [9]. In the view of the genetic result and the patient’s most recent IOP, bilateral trabeculectomies were performed to better control his IOP and minimize damage on his optic nerves.

This variant was not detected in either parent of the index case (Fig. 1b). The marker profile comparison using the AmpFLSTR® Identifiler® PCR Amplification Kit confirmed a profile consistent with the proband being the biological child of the stated parents, indicating p.(Pro254Leu) has arisen *de novo* in the proband. A *de novo MYOC* pathogenic variant, p.(Val251Ala), has been previously reported once in a JOAG case [21]. Interestingly, this variant was located three amino acids from p.(Pro254Leu) which was identified in this study.

While the occurrence of *de novo* pathogenic variants in the genome vary considerably based on genomic location, they are estimated to be common and have been linked to many sporadic diseases [22]. Conditions with dominant inheritance and modest fitness effect are more commonly inherited than caused by *de novo* variants, and this is the situation for *MYOC* associated glaucoma which is usually inherited. For example, a founder effect with an origin prior to the European settlement of Australia has been suggested for the most common *MYOC* disease-causing variant, p.Gln368Ter, in some families [23]. However, we previously reported that 20 % of *MYOC* carriers do not report a family history of the disease [9]. Although this may be explained by individuals not being aware of a diagnosis in their families, or relatives being undiagnosed, it is possible that variants occur *de novo* in some families. *MYOC* variants are often identified in older individuals with parents usually unavailable for testing, making it difficult to evaluate whether variants are inherited or sporadic. This case is the second report of a *de novo MYOC* variant, emphasizing that a sporadic variant should be considered when evaluating the likelihood of *MYOC* variants in cases with no family history of JOAG or POAG.

*De novo* variants arise either in the germline or during embryogenesis. If present in the germline cells of one parent, they can represent a recurrence risk in siblings of the variant carrier. We have previously shown that *MYOC* genetic testing is important for early identification of at-risk individuals and appropriate interventions to minimize irreversible vision loss [9, 24]. To exclude a recurrence risk resulting from germline mosaicism, both siblings of the proband were subsequently tested for the *MYOC* variant. Our testing revealed that neither sibling carried the *MYOC* p.(Pro254Leu) variant, eliminating an inherited risk of developing *MYOC* associated glaucoma.

## Conclusion

In conclusion, we report a novel *de novo MYOC* variant considered pathogenic in a patient with sporadic JOAG. This is the second report of a *MYOC de novo* variant, and it is currently unknown if this mechanism occurs more frequently. This case also highlights that *MYOC* testing should not be restricted to individuals with a positive family history of glaucoma.

### Consent

Ethics approval was obtained from the Southern Adelaide and Flinders University Clinical Research Ethics Committee. The study conformed to the tenets of the Declaration of Helsinski and follows the National Health and Medical Research Council statement of ethical conduct in research involving humans. Written informed consents were obtained from each participating family member. A copy of the written consent is available for review by the Series Editor of this journal.

## Abbreviations

ANZRAG: Australian and New Zealand Registry of Advanced Glaucoma
IOP: intraocular pressure
JOAG: juvenile open angle glaucoma
MYOC: Myocilin
NATA: National Association of Testing Authorities
POAG: primary open angle glaucoma
